# Plasma modification of graphene nanoplatelets surfaces

**DOI:** 10.1186/s11671-023-03929-y

**Published:** 2023-11-24

**Authors:** Tyler Johnson, Keliang Wang, Qi Hua Fan, Andre Lee

**Affiliations:** 1 Department of Chemical Engineering and Materials Science, Michigan State University, East Lansing, MI 48824 USA; 2 Fraunhofer USA Center Midwest, East Lansing, MI 48824 USA; 3 Department of Electrical and Computer Engineering, Michigan State University, East Lansing, MI 48824 USA

**Keywords:** Plasma, Graphene, Graphene nanoplatelets, Surface modification, Composites

## Abstract

Atmospheric plasma processing, which combines the efficacy of chemical processes and the safety of physical processes, has been used to modify the surface characteristics of graphite-based materials. In this work, two distinct plasma source gases, C_4_F_8_ and O_2_, with the addition of a rotary reactor were used. The effectiveness of modifying the basal plane of intercalated graphite nanoplatelets (GnP) was investigated with various analytical techniques and the visual observation of the dispersion of these plasma-treated GnP in solvents was also reported. It is shown that this low-temperature plasma processing technique can be used to successfully modify the GnP surface without significantly changing the intrinsic structure of the GnP, which is desirable in many applications. With the C_4_F_8_ plasma treatment, the immersion characteristics in solvents can be tuned and the functional groups present on the surface can be tailored to produce desired bonding environments. This surface chemistry tunability will provide the needed functionalities in creating graphene-containing composite materials.

**Supplementary Information:**

The online version contains supplementary material available at 10.1186/s11671-023-03929-y.

## Introduction

Graphene has remained an attractive material due to its excellent intrinsic mechanical, thermal, and electrical properties [1–3]. However, due to the chemically inert nature of the basal plane, significant engineering and/or chemical treatments of graphene are needed for it to be an effective reinforcement in composite materials [4]. The ways to incorporate the attachment points are varied, including chemical treatments and physical processes. Chemical treatments, such as the Hummers’ method to create graphene oxide [5], are batch processes. Harsh chemicals are needed to intercalate and allow oxidation, which can be a time-consuming process and results in the degradation of many properties that make graphene desirable in the first place [6]. The chemicals and reactions involved create exothermic conditions non-conducive to large-scale manufacturing. Physical processes, such as sonication in solvents [7] and mechanical grinding procedures [8], are simple and more environmentally friendly but also less effective in creating strong interfacial bonds. These physical processes can also require large amounts of energy to break bonds within the graphene and ensure an environment where those broken bonds will reform according to the desired bonding schemes [8].

Plasma treatment of graphene surfaces has been shown to successfully modify the basal plane of graphene without the creation of harsh by-products [9–12]. Plasma modification of graphene utilizes energetic and/or reactive radicals in the plasma to interact with the surface and can have several effects, such as breaking the C–C bonds, removing surface atoms, “cleaning” the surface, and modifying the surface chemistry [13]. The surface reactions are determined by the types of plasma species and energy, which depend on the plasma system, source gas and pressure, and excitation power. Oxygen plasmas have been used to increase bonding between graphene and various matrix materials. Oxygen plasma functionalized graphene has been shown to increase adhesion and stress transfer in metal composites [14]. Oxygen plasmas have also been shown to increase the functionalizability of graphene materials in general, creating more active reaction sites for further functionalization [15]. Oxygen plasma functionalization on nitrogen-doped graphene has been shown to increase supercapacitor performance by inducing pseudo-capacitance incorporating nitrogen and oxygen functional groups [16]. Other carbon-based materials, such as carbon black, have been treated with oxygen plasma; aiming to improve its dispersibility without changing intrinsic structure. Using oxygen plasma functionalization, carbon black was transformed from easily agglomerating nanoparticles to a network with high surface area and hydrophilicity, increasing its usability as a desalination agent [17]. Carbon nanotubes have been modified with plasma for various applications. Treatment with mixtures of oxygen and argon gasses have been shown to increase oxygen content and improve electrochemical sensing performance [18]. Plasma produced using mixtures of carbon dioxide and nitrogen have been shown to alter the nanotubes surface, by producing isocyanate functionalities, and increase the wear resistance of polyurethane-nanotube composites [19]. Amine functionalization using nitrogen plasma has been performed on reduced graphene oxide, with amine functionals providing enhanced interfacial bonding [20]. Nitrogen plasma has been used to produce successful biosensors using graphene as a base material [21]. Mixtures of argon and nitrogen gasses have been used to produce a plasma that can reduce and functionalize graphene oxide simultaneously [22]. Previous reports show that octafluorocyclobutane plasma can functionalize graphite, while oxygen plasma can etch single layer chemical vapor deposited graphene in a few seconds [23, 24]. In this work, radio frequency (RF) power is used to excite gases in a lab-scale plasma reactor, enabling the radicals to interact with graphene nanoplatelets (GnP). The interactions of the plasma species with the GnP create surface disorder, making it possible to introduce covalently bonded functional groups to the graphene surface.

In conventional plasma treatment systems, the sample remains static within the reaction chamber and electrodes are positioned such that the plasma plume interacts with the sample surface. This setup geometry limits the plasma interaction with the sample materials, especially powder samples, to the exposed surfaces. Depending on the energy of the radicals in the plasma, treatment of the bulk material, or at any depth below a few layers of surface atoms, becomes improbable. As radical energy increases, interactions deeper into the surface of the material become possible but implantation and ablation become issues, destroying bonds, and creating disorder within the material. In this work, we report using our patented rotary plasma reactor to overcome these limitations [25]. The rotary plasma reactor is particularly attractive for treating the surface of powders like GnP. The rotation of a tumbler will expose fresh GnP surfaces to the plasma for effective modifications. The goal of the plasma treatments is to modify the GnP surface to allow easy incorporation into solvents and composites, while not significantly changing the structure of the GnP and preserving the intrinsic properties. By utilizing the rotary reactor, lower energy plasma can be utilized to fully treat powder samples, especially large quantities of powders that are difficult to treat in conventional stationary sample set-ups. Using lower energy plasma limits the structural modifications of the GnP, preserving the intrinsic properties, and promoting chemical modification of the surface without implantation or ablation. This minimal change in the GnP structure would allow for more efficient reinforcement in composites.

To verify the plasma treatment effects, X-Ray Photoelectron Spectroscopy (XPS) was used to evaluate the surface functionalization; Raman Microscopy and X-Ray Diffraction (XRD) were used to monitor structural changes of the GnP; and immersion experiments were used to qualitatively visualize the treatment uniformity of the sample batch..

## Materials and methods

Graphene Nanoplatelets (GnP) of grade M25 were supplied by XG Sciences and used as received. According to XG Sciences, this grade of GnP was produced using a mechanical milling process of acid-assisted intercalated graphite powders. This process is known to leave residual species from the acid intercalation, including oxygen containing groups. These residual oxygen groups are characterized in the raw materials XPS spectra. Grade M particles have thicknesses of approximately 6–8 nm and a surface area of 120–150 m^2^/g, with M25 having an average particle diameter of 25 microns [26].

**Fig. 1:**
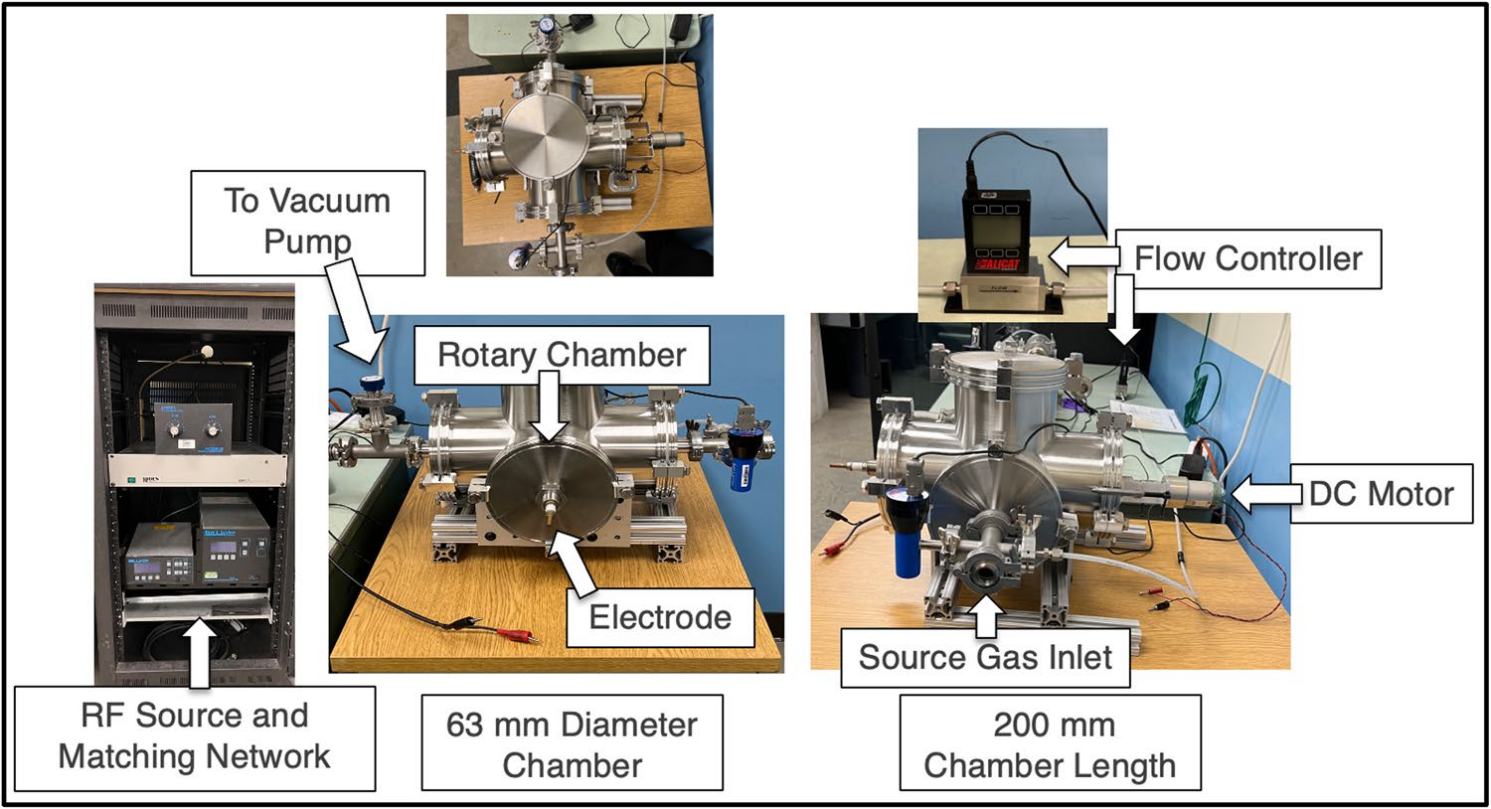
Annotated layouts of the rotary plasma reactor

**Fig. 2:**
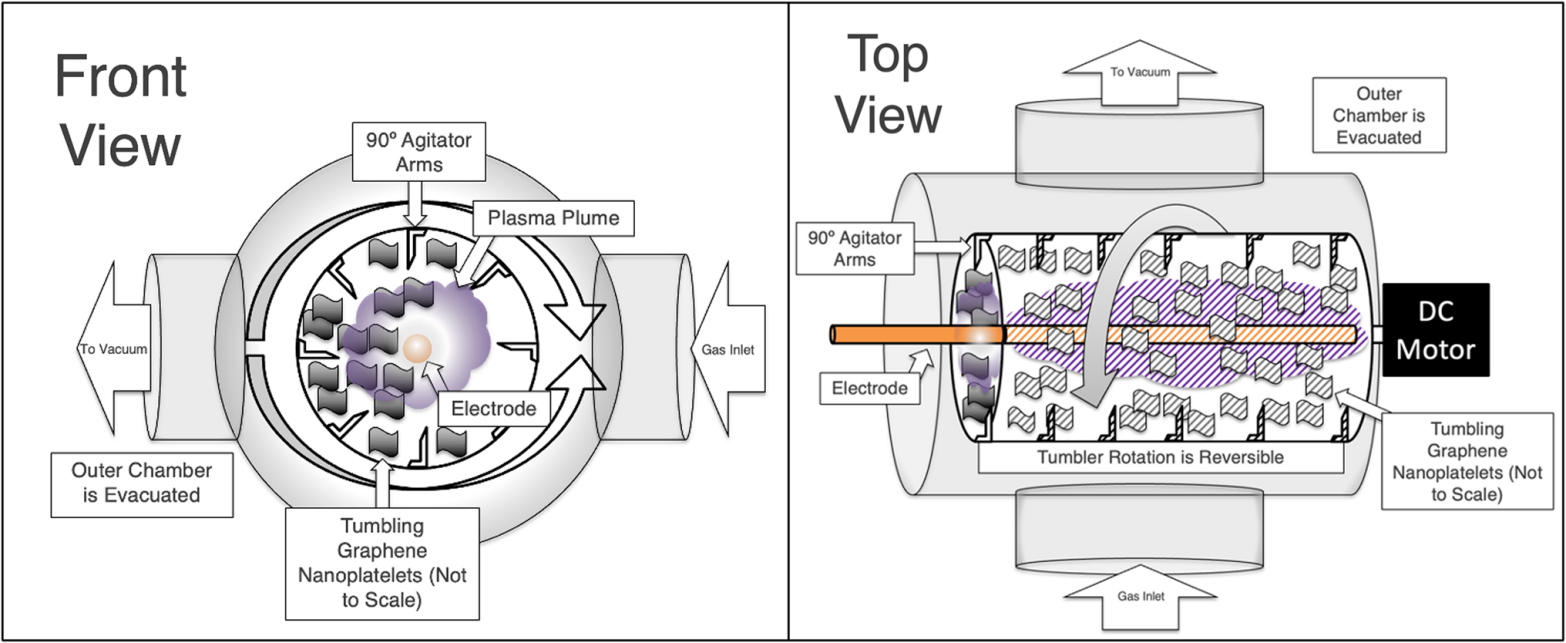
Schematic of the rotary plasma reactor showing electrode placement and inner reaction tumbler

A radio frequency plasma system was used to treat the GnP (Fig. 1). The plasma system included a rotary tumbler chamber of 63 mm diameter and 200 mm length that served as the vacuum chamber, a mechanical vacuum pump, a 13.56 MHz RF power supply, a matching network, a vacuum gauge, and a gas flow controller. A schematic of the inner tumbler shows electrode location and tumbling of sample materials (Fig. 2).

First, 1 g of M25 GnP was loaded into the rotary tumbler and the chamber was evacuated to a base pressure of < 20 mTorr. Although no high-vacuum pump was used, the reactor was constructed leak-tight by using magnetic feedthrough for the rotary components. Then the chamber was purged five times with the working gas prior to each plasma treatment. The effects of residual oxygen and water was minimized as evidenced by the consistent results obtained with multiple samples from each treatment as well as from different batches. Next, the gas flow rate was adjusted such that the pressure was stable at 660–680 mTorr (87.9–90.7 Pa), this was achieved at a flow rate of 8 standard cubic centimeters per minute. Octafluorocyclobutane (C_4_F_8_) and oxygen (O_2_) were used separately to generate plasmas and treat samples. Samples treated with C_4_F_8_ plasma are referred to as C_4_F_8_ Plasma treated M25, samples treated with O_2_ plasma are referred to as O_2_ Plasma treated M25. Untreated powders, which were used as-received for comparison, are referred to as As-Received M25. Next RF power was turned on and the matching network was tuned manually to minimize reflected power. The RF power was set at 70 W in all the treatments. The samples were treated for 10 min with reversing rotation direction every 30 s to move the GnP back and forth from one end to the other for uniform treatment. Standard gas flow control and pumping system were used to handle the C4F8 gas and exhaust in an effective venting environment. The components that are directly exposed to the C4F8 plasma were made of aluminum, while the other system components were made of stainless steel. No damage to the reactor components was observed.

X-ray Photoelectron spectroscopy was carried out using a Perkin Elmer Phi 5600 ESCA system with a magnesium Kα x-ray source at a take-off angle of 45°. Survey scans were taken at 1.6 eV step and 187 eV pass energy, detailed region scans were performed with 0.05 eV step and 11.75 eV pass energy. The fitting of peaks was performed using CasaXPS software.

Raman spectroscopy was carried out using LabRAM ARAMIS (HORIBA JOBIN YVON, Inc) in the range of 1200–3000 cm^−1^ with a 50 × objective lens. The excitation laser wavelength was 532 nm. Scans were performed using 1800 gr/mm grating with a 180-s accumulation time.

X-ray diffraction was carried out using a Bruker D2 Phaser (Bruker Corporation). Scans were collected at 30 kV and 10 mA with a 0.01° step size and 0.5 s dwell time.

Immersion experiments were carried out using deionized water and isopropyl alcohol. Approximately 20 mg of sample materials were added to approximately 60 mL of liquid. As-Received M25, C_4_F_8_ Plasma treated M25, and O_2_ Plasma treated M25 were used to compare how surface treatment affected behavior in water and alcohol. Samples were weighed, then added to a beaker containing the liquid and manually stirred with a glass stir rod. The video recording of the experiments is available in Online Resource 1 and Online Resource 2.

## Results and discussion

**Fig. 3:**
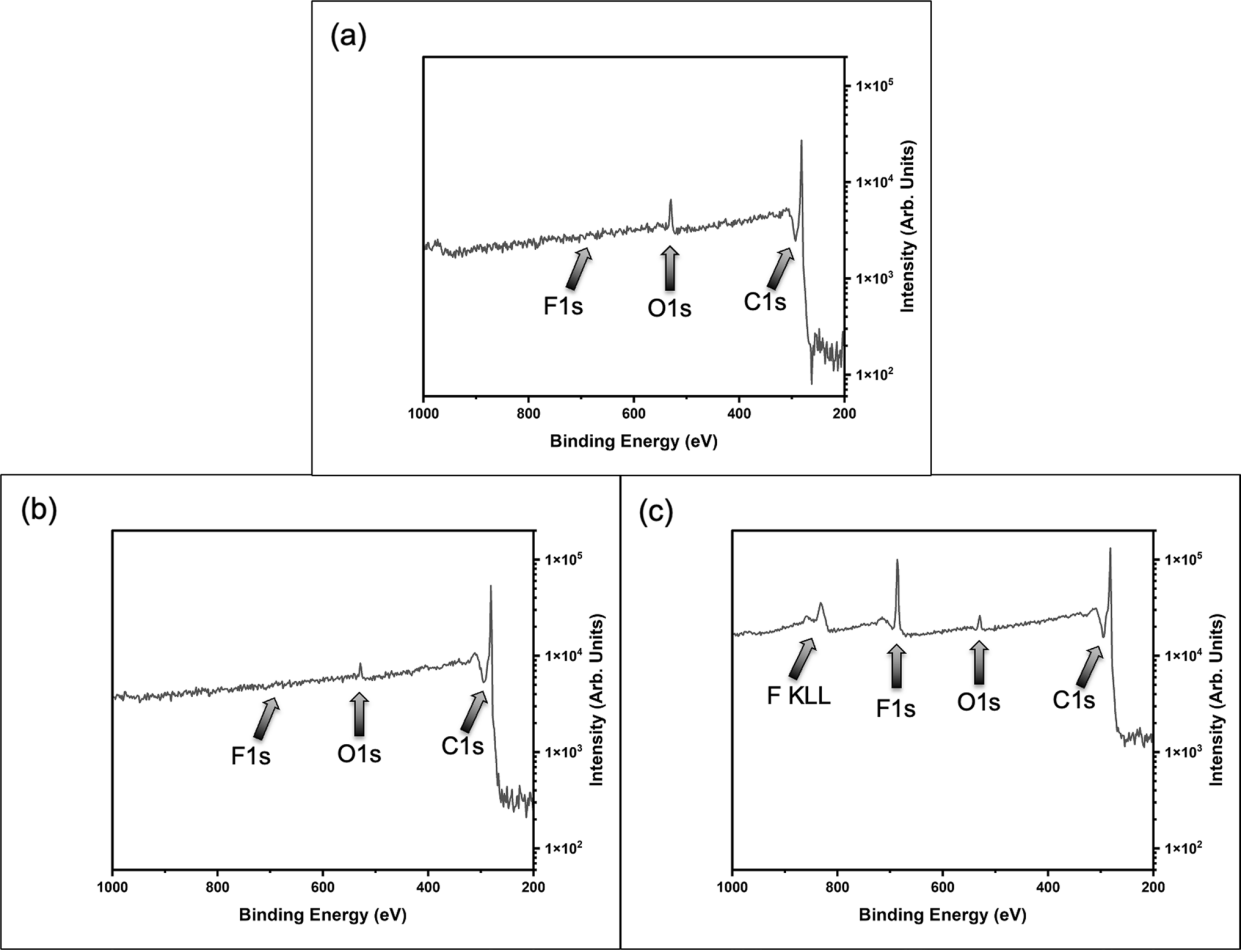
XPS survey scans **a** As-Received M25, **b** O_2_ Plasma treated M25, and **c** C4F8 Plasma treated M25. Peaks of interest are annotated

**Fig. 4:**
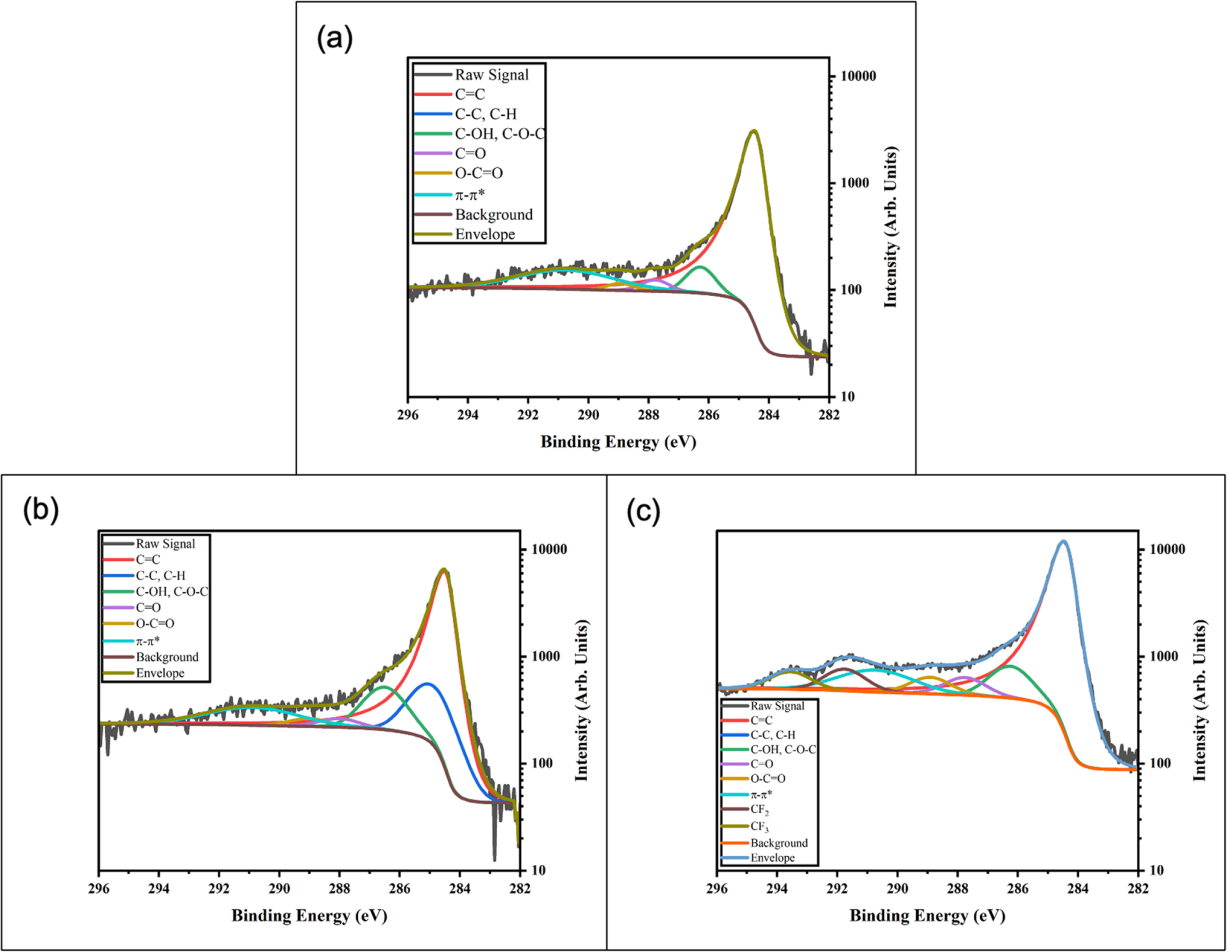
Detailed XPS scans of the C1s region for **a** As-Received M25, **b** O_2_ Plasma treated M25, and **c** C4F8 Plasma treated M25

Initial XPS survey scans were performed to identify areas of interest for detailed XPS scans (Fig. 3). Survey scans of the raw materials (Fig. 3a) show only C1s and O1s peaks. We expect to see some residual oxygen species from the GnP exfoliation process, these species are characterized in the detailed C1s scans (Fig. 4a). No detailed scans were performed in the F1s region as no peaks were identified in the survey scans. Oxygen plasma treated materials show a similar survey spectrum to that of the raw materials (Fig. 3b). Detailed scans were performed in the C1s and O1s regions for As-Received M25 and O_2_ Plasma treated M25. The C_4_F_8_ Plasma treated M25 showed C1s and O1s peaks, but also displayed an F1s peak and an associated peak corresponding to the fluorine auger process (F KLL) (Fig. 3c). Detailed scans were performed in the C1s, O1s, and F1s regions for C_4_F_8_ Plasma treated M25.

Peak fitting of XPS spectrum was performed using CasaXPS software. Background subtraction was performed, and spectra were charge-referenced to the 284.5 eV C=C peak. A general fitting scheme as suggested by Biesinger [27] for graphitic/graphene/carbon nanotube type materials was modified and used. This fitting scheme was applied to the C1s spectrum from As-Received M25 (Fig. 4a). Full width half max (FWHM) restrictions for the shake-up peak, π–π*, were relaxed to 3.47 eV to allow broadening of the peak to better fit experimental data. The details of the peak assignments and constraints are outlined in Table 1.

**Table 1:** Constraints used in CasaXPS software to fit XPS spectra [28]

Species	Peak	Starting Position (eV)	Common Range (eV)	FWHM (eV)	Lineshape	Area Constraint
C=C	A	284.5		0.4–0.8	LA (1.2, 2.5, 5)	
C–C, C–H	B	285	284.8–285	0.9–1.5	GL (30)	
C–OH, C–O–C	C	286.5	286.3–286.7	0.9–1.5	GL (30)	
C=O	D	288	287.8–288	0.9–1.5	GL (30)	
O–C=O	E	289	288.8–289.3	0.9–1.5	GL (30)	
Pi to Pi*	F	290.91		2.7	GL (30)	A*0.06963
C-F2	G	291.2 [28]		0.9–1.5	GL (30)	
C-F3	H	293.1 [28]		0.9–1.5	GL (30)	

This fitting scheme is an appropriate starting point for most graphitic materials. The C=C peak will be the most prominent peak and can be used for charge correction [27]. Adventitious carbon, while useful for samples where other known charge referencing peaks are not available, should be treated differently than graphitic carbon [27]. We are investigating graphene nanoplatelets, so it is expected there will be a large graphitic C1s peak at 284.5 eV, and a large π–π* peak contribution. Any adventitious carbon will be captured in the 285 eV peak, C–C or C–H, and can be characterized as such, but should not be used for charge correction in this case [27]. Data from XG Sciences indicates the presence of ethers, hydroxyl, and carboxyl groups due to residuals from the intercalation process, these are accounted for and characterized in the starting material. Fitting of peaks around 4–6 cm^−1^ shifts from C=C can be complicated due to overlapping oxygen and fluorine functional group peaks in this region. Notably, O–C=O and C–F can be convoluted and will appear in the same region. This fitting scheme was then applied to spectrum taken from treated materials. When applying the general fitting scheme to the fluorine plasma treated spectrum, two additional peaks are seen (Fig. 4b).

These peaks are a result of the plasma treatment process and are assigned accordingly. The assignment of the CF_2_ and CF_3_ peaks are in good agreement with literature values for energy shifting [28]. We do not expect to see many C-F type functionals due to the structure of the C4F8 molecule. It is more likely the ionization process of C_4_F_8_ causes a breakdown leading to abundance of CF_2_ type radicals [29]. However, these C-F functionals would overlap near the π–π* peak and the O–C=O peak. Therefore, broadening and increases in intensity in 290–293 eV region could be due to fluorine functionalization. Oxygen groups, from the residuals left after XG Sciences production process and from quenched reaction sites, are present in the C_4_F_8_ Plasma treated M25. When radicals from the plasma interact with the GnP surface, active sites are created through disorder. When the sample materials are removed from vacuum, these sites react with the environment and are passivated, usually through reactions with water producing hydroxyls.

Fitting of the oxygen plasma treated samples did not show any additional peaks, only increases in intensity for the oxygen functional peaks (Fig. 4c).

**Fig. 5:**
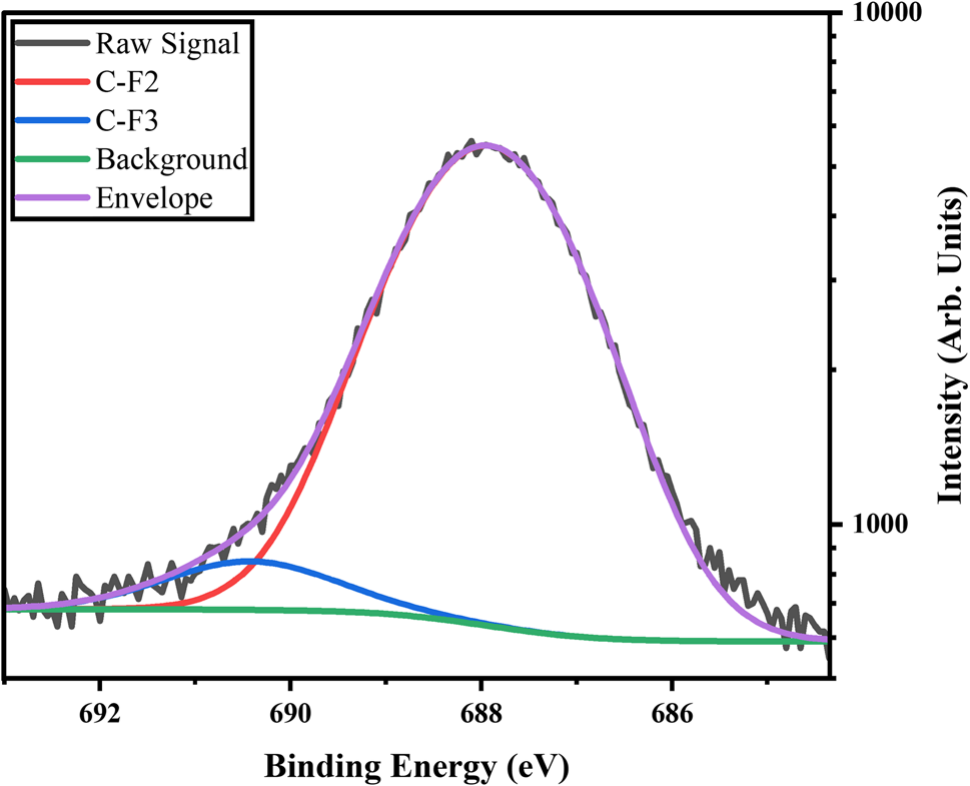
XPS Spectrum of the F1s region of C4F8 Plasma treated M25. Peaks were fit using CasaXPS software

Fitting of the F1s peak for fluorine plasma treated samples can only be accomplished by fitting with two or more component peaks, shown in Fig. 5 for the C_4_F_8_ plasma treated samples.

**Fig. 6:**
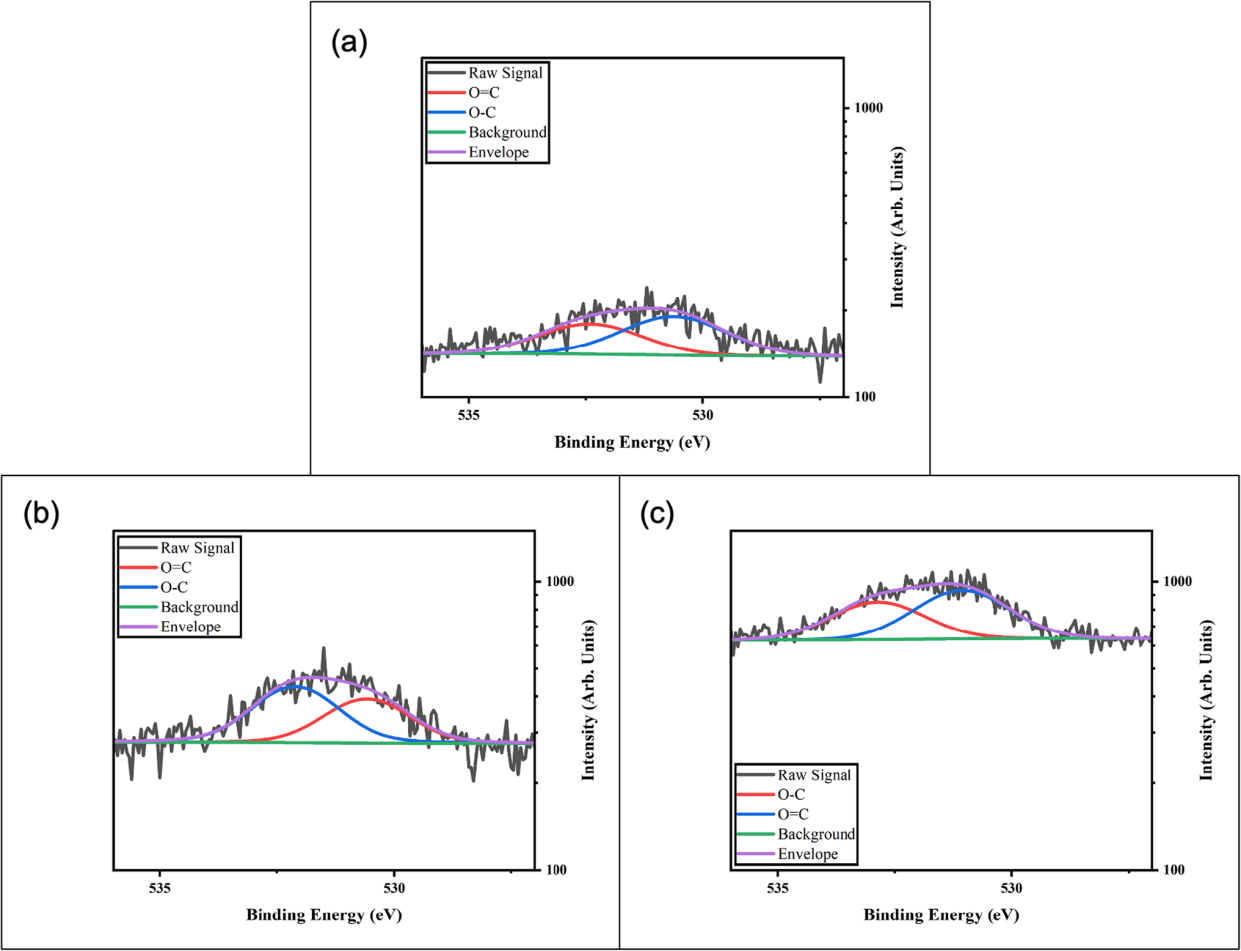
Detailed XPS scans of the O1s region for **a** As-Received M25, **b** O_2_ Plasma treated M25, and **c** C_4_F_8_ Plasma treated M25

These results support that more than one type of fluorine species is present at the surface of the GnP after plasma modification. We expect to fit with at least two peaks due to the two extra peaks in Fig. 4b, however due to overlaps in the C1s spectrum of oxygen and fluorine bonded carbons there may be additional peaks convoluting the F1s spectrum. The fluorine treatment did reduce the oxygen content from 3.31 at% in the as received GnP to 2.0 at% after the 10-min fluorine plasma treatment. Peaks at lower binding energies are more indicative of ionically bonded fluorine (< 688 eV), while higher binding energy peaks are more covalent in nature [23, 28]. The peak at 688 eV indicates mainly covalently bonded fluorine, but shoulders can be fit to this peak reducing the residual standard deviation of the fit. This could indicate multiple bonding environments for the fluorine, which would be expected due to the deviation in energy of the radicals encountering the surface. Further treatments should be conducted to study the evolution of the F1s peak to better understand the changing bonding environment. Notably, fluorine peaks are not present in the XPS spectra of the raw material, or the oxygen plasma treated material. Peak fitting of the O1s peaks for oxygen plasma treated samples leads to two peaks being identified (Fig. 6b).

These peaks can be roughly associated with a double bonded oxygen in a carbonyl type carbon group and a single bonded oxygen in a carboxylic type of carbon group [30]. These double bonded carbonyl type oxygen peaks will be located at low eV values (530–533 eV) while single bonded carboxylic type oxygen peaks will be located at high eV values (533–537 eV). These peaks could be further deconvoluted with additional peak assignments, however over-fitting becomes a concern due to the signal to noise ratio. The XPS O1s spectrum for As-Received M25 (Fig. 6a) shows two peaks, one at 530.6 eV and the other at 532.4 eV. Comparing the spectra for As-Received M25 and oxygen plasma treated M25, there was an increase in the ratio of single bonded oxygen species. This supports the evidence from Fig. 4c which shows an increase in the C–H and C–OH peaks as compared to the raw spectrum in Fig. 4a; and a dampening of the C=O and O–C=O peaks. This change in ratio indicates addition of preferable single-bonded oxygen groups as compared to hard to functionalize double-bonded oxygen groups. Notably, the peak positions for oxygen plasma treated samples do not change significantly from that of the raw material. Peaks for the oxygen treated samples were located at 530.6 eV and 532.1 eV, respectively. This indicates that oxygen species present are not changing, only the ratio of the species present. Overall oxygen content has increased for oxygen plasma treatments as compared to the As-Received M25. The density of oxygen increased from 3.31 at% in the as-received GnP to 5.74 at% with 10-min oxygen plasma treatment.

Like the other two samples, the O1s XPS region for C_4_F_8_ plasma treated samples can also be fit with two peaks (Fig. 6c). Compared to the untreated sample, both peaks were shifted to slightly higher binding energies, one at 531.1 eV and one at 532.9 eV, respectively. This could indicate the oxygen species present are becoming more carboxylic and single-bonded in nature, or the oxygen species are changing. Fluorine plasma treatments resulted in an oxygen content of 2.0 at% at 10 min. These results indicate that fluorine plasma treatments are either removing oxygen species through sputtering or are functionalizing these oxygen attachment points; changing the binding energies.

Raman microscopy is a powerful tool for the structural characterization of carbon materials [31–34]. Raman microscopy results show changes in the spectrum for different plasma treatments.

**Fig. 7:**
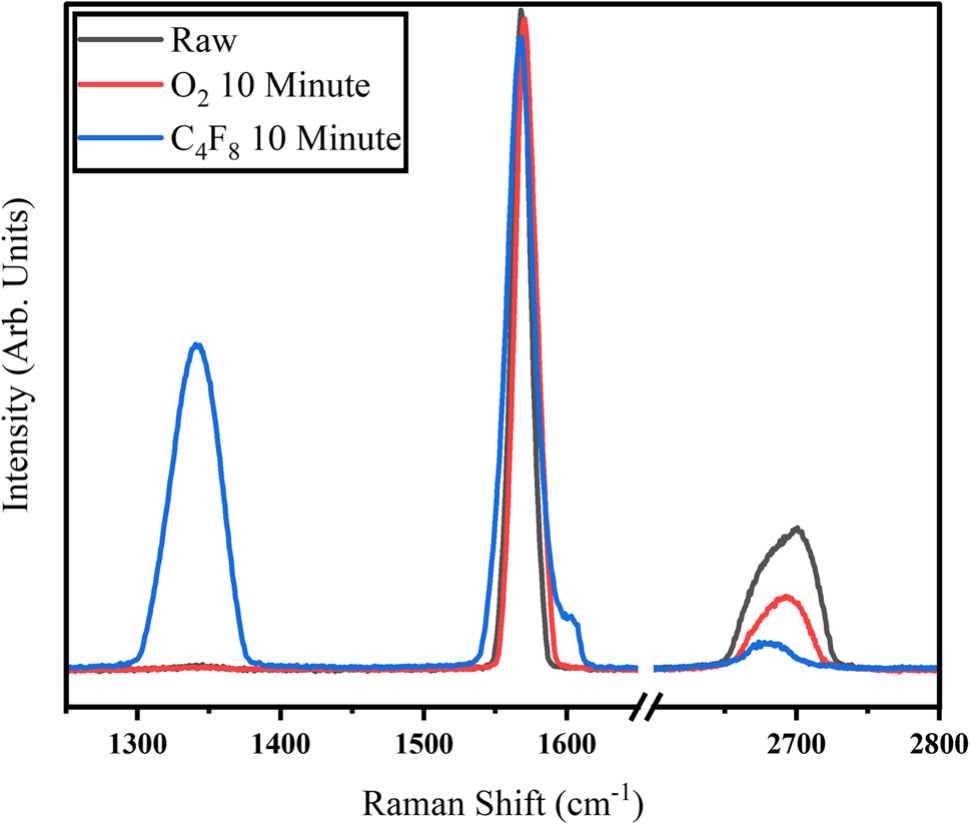
Raman spectrum for samples comparing As-Received M25, 10 Minute O_2_ Plasma, and 10 Minute C4F8 Plasma samples

Table 2 summarizes approximate peak position and intensities; these were used to find ratios between peak intensities. The only spectrum containing a D-Peak is the C_4_F_8_ plasma treated samples (Fig. 7).

**Table 2:** Table summarizing Raman peak position and intensities used to calculate intensity ratios

Sample	D-Peak	G-Peak	D′-Peak	2D-Peak
M25 Raw	N/A	1568 cm^−1^ 38,773 cnts	N/A	2700 cm^−1^ 8417 cnts
M25 O_2_ 10 min	N/A	1571 cm^−1^ 38,243 cnts	N/A	2693 cm^−1^ 4426 cnts
M25 C_4_F_8_ 10 min	1342 cm^−1^ 18,996 cnts	1568 cm^−1^ 37,162 cnts	1605 cm^−1^ 2870 cnts	2680 cm^−1^ 1633 cnts

The D-Peak at around 1350 cm^−1^ is representative of breaking the symmetry of the carbon basal plane by breaking of pi bonded carbons and allows non-symmetrical stretching, a transverse zone boundary phonon interacts with defect to create this shift. The D-Peak can be used to track the amount of “disorder” of the basal plane, specifically the intensity of the D-Peak with respect to the G-Peak [35]. The G-Peak at around 1580 cm^−1^ represents the symmetric “breathing” of the perfect carbon basal plane, perfect graphene will contain only a G-Peak and a G′ peak. The C_4_F_8_ plasma treatment resulted in an I_d_:I_g_ of 0.51, which is significantly higher than that of the As-Received M25 and the O_2_ treated M25 samples. The oxygen plasma treatments did not create an appreciable D-Peak, meaning there is relatively little basal plane disorder. This could be indicative of the addition of functional groups mainly at the edges of the GnP sheets. This is supported by literature from XG sciences indicating that their GnP will be more readily functionalized at the edges [36]. The similarity between the raw material spectrum and the oxygen plasma treated material spectrum indicate no structural changes are occurring during the oxygen plasma treatment, only an increase in the oxygen functionals at the edges of the sheets.

Also seen in the spectrum of the C_4_F_8_ treated samples (Fig. 7) is a shoulder at approximately 1605 cm^−1^, this represents the D′-Peak. The D′-Peak is an intravalley double resonance process where a longitudinal phonon interacts with a defect in a circle around an equivalent k point in the Brillion zone. This is unlike the G′-Peak, which is intervalley, meaning around nonequivalent k points and is a result of two longitudinal phonons interacting, no defects are required to produce a G′-Peak which is why it is present in perfect graphene spectra. Presence of the D′-Peak is further evidence of defects generated during the C_4_F_8_ plasma treatment process. The G′-Peak has been shown to be sensitive to both doping and strain in graphene, as well as an indication of the number of layers of graphene with AB Bernal stacking [33]. Decreasing the layer count in few-layer graphene will lead to a redshift in the G′-Peak, increasing strain will tend to redshift the G’-Peak, increasing doping will lead to a dampening in the peak intensity. Graphene produced through mechanical exfoliation will usually have predominantly AB stacking, however the sample material used here was subjected to unknown chemical processes which may have altered the stacking order and produced rotationally random graphene platelet agglomerates. In this case, the G′-Peak could be used to infer about double resonant selection rule relaxation associated with the random stacking [33]. We observe that plasma treatment both redshifts and dampens the G’-Peak (Fig. 7); with fluorine plasma creating more shifting and dampening. This is consistent with the results for the I_d_:I_g_ ratio indicating that more disorder was created in the C_4_F_8_ treated samples. The peak shape is also changed from a Lorentzian type of peak shape for raw materials to a broader, more convoluted Lorentzian for C_4_F_8_ treated samples.

**Fig. 8:**
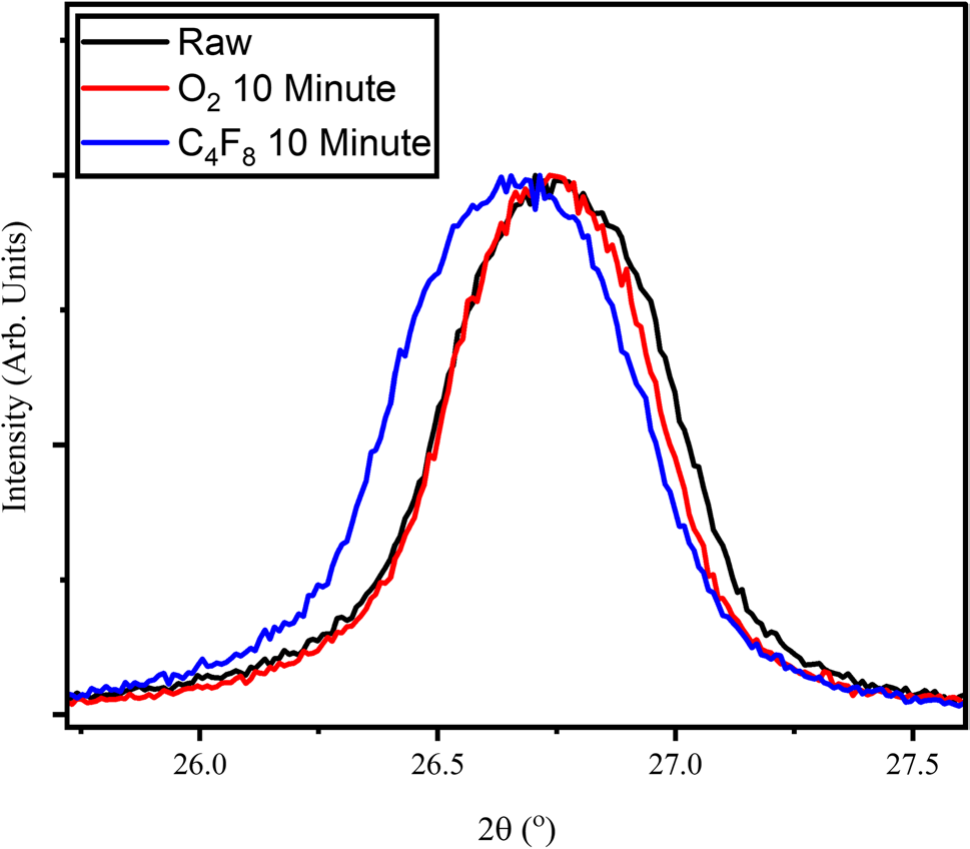
X-Ray Diffraction results for As-Received M25, 10 Minute O_2_ Plasma, and 10 Minute C4F8 Plasma. Intensity has been normalized

X-ray diffraction can be used in conjunction with Raman to help determine the crystallite size in the c-axis [33]. Diffraction scans are compared in Fig. 8, As-Received M25 samples show a peak at 26.75° with decreasing diffraction angles as treatment occurs. Peak positions, intensities, and full width half maxes (FWHM) are reported in Table 3.

**Table 3:** Table summarizing the peak position, intensity, and full-width at half maximum of the X-Ray Diffraction results

Sample	Diffraction Angle (°2$\theta$)	Intensity (counts)	FWHM (°2$\theta$)
M25 Raw	26.75	4355	0.55
M25 O_2_ 10 min	26.74	4565	0.51
M25 C_4_F_8_ 10 min	26.67	3831	0.61

By deconvoluting the G’-Peak and comparing results to XRD scans, empirical formulas have been developed to determine this c-axis crystallite size from certain Raman parameters, R, which is a ratio of the intensities of the deconvoluted G′-Peak. Essentially, the relationship found is that as the G′-Peak becomes more skewed to higher Raman shifts, the crystallite size should increase. From Fig. 7, we see that the As-Received M25 material is the most blue-shifted peak and as we treat with plasma the peak redshifts and changes shape. This is supported by diffraction results (Fig. 8), where the shifting to lower 2$\theta$ and broadening of the peak FWHM indicates a smaller crystallite size according to the Scherrer equation. Plasma has been shown to exfoliate graphene under certain conditions [37]. Additionally, the plasma processing combined with mechanical tumbling may increase the likelihood of exfoliation. The slight shifting coupled with the increases in FWHM could indicate a peeling of layers due to plasma interactions. The slight shifting could indicate a larger inter-galley spacing for the GnP, but this effect is negligible as the d-spacing remains 0.33 nm.

These results indicate there is minimal structural change occurring to the platelets during plasma treatments. Structural characterization of these platelets will become important when used in composite applications with these functionalized GnP nanoplatelets. For this preliminary study, we are more concerned with the surface chemistry present on these functionalized platelets. Further structural characterization in composites will be the subject of subsequent studies exploring the applications of these functionalized GnP nanoplatelets.

**Fig. 9:**
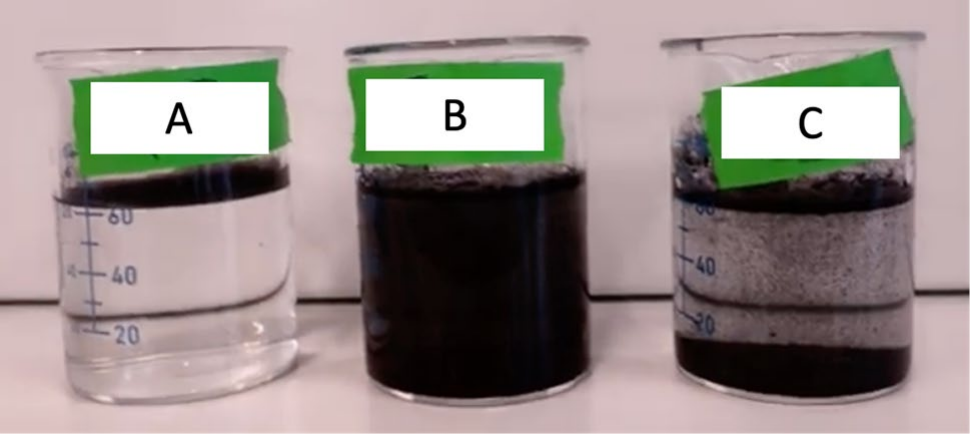
Still image of immersion experiments, 20 mg of GnP in 60 ml of DI Water. A is C4F8 treated M25, B is O2 treated M25, and C is As-Received M25

**Fig. 10:**
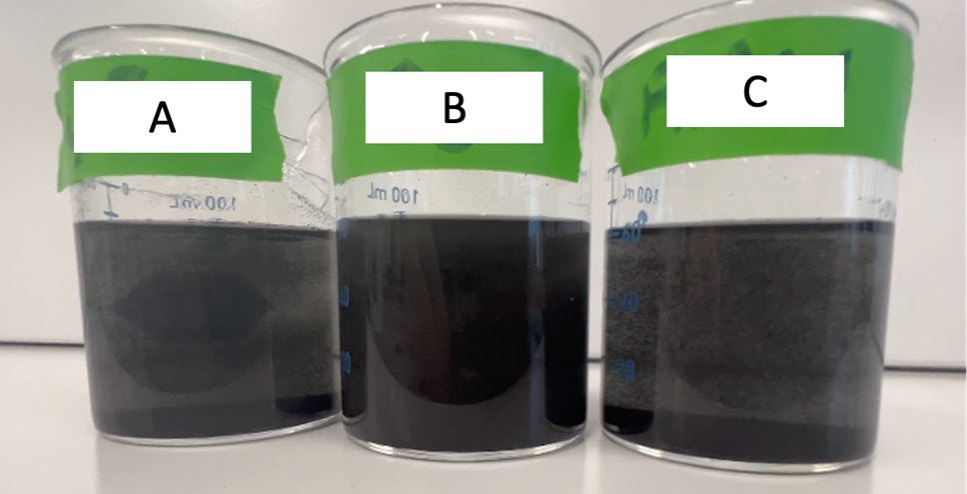
Still image of immersion experiments, 20 mg of GnP in 60 ml of Isopropyl Alcohol. A is C4F8 treated M25, B is O_2_ treated M25, and C is As-Received M25

For composite applications, in addition to surface chemistry of the platelet, dispersion of these functionalized GnP nanoplatelets is critical for effective matrix reinforcement [38]. Therefore, immersion experiments were conducted to observe how the plasma treatments changed dispersion behavior in deionized (DI) water and isopropyl alcohol. Images of the immersion experiments are displayed in Figs. 9 and 10, qualitative inspection can be performed. The video recording of the immersion experiments is available in Online Resource 1 and Online Resource 2.

Results of these experiments show that fluorine surface treatments increase the hydrophobicity of the M25 GnP significantly. As seen in Fig. 9, the C_4_F_8_ 10 Minute Plasma sample does not disperse in the DI water. Increased hydrophobicity is an indicator of the presence of C–F bonds on the surface of graphene [28, 39]. Dispersibility in isopropyl alcohol is comparable to untreated GnP for the fluorine treated samples. Shown in Fig. 10, there is little difference in the appearance of the solutions, with the oxygen plasma sample being slightly more dispersed by visual inspection. Oxygen plasma treated samples show an increase in dispersibility for both water and isopropyl alcohol over nontreated samples. The increase dispersibility in both water and isopropyl alcohol are due to the increased disorder on the basal plane of graphene and presence of oxygen containing functional groups.

## Conclusions

In this work, commercially available graphene nanoplatelets were modified with low-temperature plasma processes. These plasma processes were performed in a patented rotary reactor system which allowed for increased treatment uniformity for powder samples. The reactor could not only move the powder up and down but also simultaneously transport it through the reactor back and forth using a set of proprietary tumblers. This led to uniform treatment of the samples. Samples randomly taken from each batch, as well as from different batches, showed almost identical Raman spectra. It is worth noting that samples from a static reactor used previously showed pronounced variations in the immersion behaviors, indicating less effective plasma treatment of the stationary powders. Modifications were characterized using XPS, Raman microscopy, and XRD. Immersion experiments were conducted to observe the materials behavior in solvent. All results indicate that C_4_F_8_ plasma treatments were successful in attachments of fluorine containing functional groups on the surface of GnP. Addition of F1s peaks on the XPS scans indicate addition of fluorine to the surface of GnP, which modifies the immersion characteristics of the GnP in water and alcohol. Fluorine atomic percentage increased from zero in the as-received materials to 12.6 at% after a 10-min C_4_F_8_ plasma treatment. Oxygen plasma treatments were successful in the addition of oxygen containing functionals to the GnP without creation of basal plane disorder. X-ray diffraction and Raman experiments show no appreciable change in the structure of the GnP, which is critical for maintaining the desirable properties of the GnP. These results indicate most oxygen functionalization is occurring on the edges of the GnP, where natural disorder provides dangling bonds. Changes to the interaction of the GnP with solvents is further evidence of a functionalized surface. Surface modification of graphene and graphene-like materials are critical for their future as composite filler materials. Through oxygen functionalization graphene materials can be made more hydrophilic and increase their dispersibility in a variety of solvents. Good dispersion of filler particles is a critical factor in creating efficiently reinforced, high-performance composites. Through fluorine functionalization, these same materials can be made more hydrophobic, creating opportunities for amphiphobicity and biosensing applications. Additionally, fluorinated graphene shows potential in energy conversion and storage devices [39]. This rotary plasma treatment provides enhanced treatment uniformity and throughput capabilities for producing these materials.

### Supplementary Information

**Additional file 1**. This video shows immersion experiments of the as-received M25 (or Raw M25), the O_2_ Plasma treated M25, and the C_4_F_8_ Plasma treated M25 in water. The video shows the three immersion experiments back-to-back with an inset of the top-down view of experiments. At the end of the video, a side-by-side comparison of the three suspensions is shown. Still images were taken from this side-by-side comparison and shown in the manuscript.

**Additional file 2**. This video shows immersion experiments of the as-received M25 (or Raw M25), the O_2_ Plasma treated M25, and the C_4_F_8_ Plasma treated M25 in Isopropyl Alcohol (IPA). The video shows the three immersion experiments back-to-back with an inset of the top-down view of experiments. At the end of the video, a side-by-side comparison of the three suspensions is shown. Still images were taken from this side-by-side comparison and shown in the manuscript.

## Data Availability

Raw data as well as plasma processed materials are available upon request.
